# The Relationship Between Exercise Habits and Stress Among Individuals With Access to Internet-Connected Home Fitness Equipment: Single-Group Prospective Analysis

**DOI:** 10.2196/41877

**Published:** 2023-02-08

**Authors:** Margaret Schneider, Amanda Woodworth, Milad Asgari Mehrabadi

**Affiliations:** 1 Institute for Clinical and Translational Science University of California, Irvine Irvine, CA United States; 2 Department of Electrical Engineering and Computer Science University of California, Irvine Irvine, CA United States

**Keywords:** stress, exercise, internet-connected home fitness equipment, physical activity, healthcare cost, health care, psychological well-being, COVID-19, online survey, user data, Peloton

## Abstract

**Background:**

Physical activity (PA) confers numerous benefits to health and health care costs, yet most adults are not meeting recommended PA guidelines. Stress may be a factor that influences PA behavior. Research investigating the impact of stress on PA has yielded inconsistent findings. Most studies find that stress negatively impacts PA, but there is some evidence that habitual exercising buffers this association.

**Objective:**

This study aims to examine the relationship between stress and exercise habits among habitual exercisers with internet-connected home fitness equipment (Peloton Bike) during the COVID-19 lockdown.

**Methods:**

Participants were recruited through Facebook (N=146) and asked to complete an internet-based survey that assessed COVID-19–related stressors, perceived stress associated with those stressors, and general perceived stress. Self-reported exercise was assessed on the survey using the Godin Leisure-time Exercise Questionnaire (GLTEQ). Participants were also asked for consent to access their Peloton usage data through the Peloton platform. From their usage data, the frequency and duration of cycling classes was calculated for 4 weeks prior to and 12 weeks following the survey. Hierarchical regression equations tested the association between stress reported on the survey and subsequent exercise participation. Exercise participation was quantified both as the frequency and duration of Peloton cycling over the 12 weeks following the survey and as self-reported moderate to vigorous activity on a second survey completed by a subset of participants 12 weeks after the initial survey.

**Results:**

There were 146 participants in our Peloton analysis sample and 66 in the self-reported exercise analysis. Peloton user data showed that study participants cycled frequently (mean 5.9 times per week) in the month prior to the initial survey, and that presurvey Peloton use was a strong predictor of exercise frequency (*R*^2^=0.57; *F*_2,143_=95.27; *P*<.001) and duration (*R*^2^=0.58; *F*_2,143_=102.58; *P*<.001) for the 12 subsequent weeks. Self-reported overall exercise likewise showed that this sample was very active, with an average of more than 8 times per week of moderate to vigorous exercise at the initial survey. Self-reported exercise on the initial survey was a strong predictor of self-reported exercise 12 weeks later (*R*^2^=0.31; *F*_1,64_=29.03; *P*<.001). Perceived stress did not impact Peloton cycling duration or frequency (*P*=.81 and .76, respectively) or self-reported exercise (*P*=.28).

**Conclusions:**

The results suggest that stress did not negatively impact exercise participation among habitually active adults with access to internet-connected home fitness equipment. Habitual exercise may buffer the impact of stress on participation in regular moderate to vigorous activity. Future research should examine the role that the availability of home-based internet-connected exercise equipment may play in this buffering.

## Introduction

It is well established that physical activity (PA) offers many health benefits including improved muscular strength [1,2], cardiorespiratory fitness [3], and mental health [4]. There is also evidence supporting PA as an effective management or preventative behavior for chronic diseases, such as type 2 diabetes [5], obesity [6-8], depression [9,10], and multiple sclerosis [11,12]. There are also financial benefits associated with PA. A study of Medicare claims among 21,750 older adults found that consistent PA throughout the lifespan was associated with lower health care costs [13]. Despite the supporting evidence for the benefits of PA, 47.3% of US adults do not meet the recommended PA guidelines for aerobic PA and even fewer meet the guidelines for both aerobic and muscle-strengthening activity (23.2%) [14].

Among the many factors that influence participation in PA, stress plays a complex role, with many studies indicating that stress negatively impacts PA participation, and a few suggesting that this association is absent or even inverted among habitual exercisers. Exercise has been defined as a subset of PA that is planned, structured, and repetitive and has as a final or an intermediate objective the improvement or maintenance of physical fitness [15]. Perhaps because of the planned and habitual aspects of exercise, it may be more resilient in times of stress as than PA, which has been defined as any bodily movement produced by skeletal muscles that results in energy expenditure [15].

Almost three-fourths of 168 studies on the stress-PA association reviewed by Stults-Kolehmainen and Sinha [16] provided evidence that higher stress is associated with less PA, whereas 17% of the studies reviewed documented increased PA during times of stress. It is worth noting that 65% of the studies documenting increased PA during times of stress actually assessed exercise, and all of these studies relied on self-report for assessments of PA and exercise. In one of the few prospective studies, the association between stress and exercise among college-aged women with the intention to exercise yielded evidence of a stress-exercise interaction such that women who were habitual exercisers tended to increase activity at higher stress levels [17]. Similar to the findings of the 2014 review, a number of reviews of studies examining changes in PA during the COVID-19 pandemic found that population levels of PA decreased [18-22]. A few individual studies, however, found that habitual exercisers increased their exercise during the lockdown [23-25]. All 3 of these studies relied on internet-based surveys to collect self-reported information on exercise. This study leveraged the data automatically archived by an internet-connected home-workout cycle (the Peloton Bike [26]) to test the hypothesis that stress would not negatively impact exercise among habitual exercisers.

## Methods

### Recruitment and Procedure

Invitations to participate in a study of Peloton Bike use and health were posted to 2 Facebook pages devoted to discussion of the Peloton Bike for 4 weeks during December 2020 to January 2021, during the time that many US residents were living under a stay-at-home order. For example, on December 3, 2020, the California Department of Public Health issued a stay-at-home order [27]. Reflecting a decline in new COVID-19 cases in January, the order was lifted on January 25, 2021. The period of data collection for this study corresponded to the holiday season, including Christmas and New Year’s Eve—a time normally typified by family gatherings, travel, and resolutions to adopt new behaviors.

A link in the invitation posted to Facebook directed potential participants to an internet-based survey. Within the survey, they provided consent to participate in the research and completed a survey on Peloton Bike use and health. Respondents were also asked to provide their Peloton username and permission for the study to access their archived usage data from the Peloton platform. Surveys were administered using the Research Electronic Data Capture (REDCap) tool [28,29]. REDCap is a secure, web-based software platform designed to support data capture for research studies, providing (1) an intuitive interface for validated data capture, (2) audit trails for tracking data manipulation and export procedures, (3) automated export procedures for seamless data downloads to common statistical packages, and (4) procedures for data integration and interoperability with external sources.

**Figure 1:**
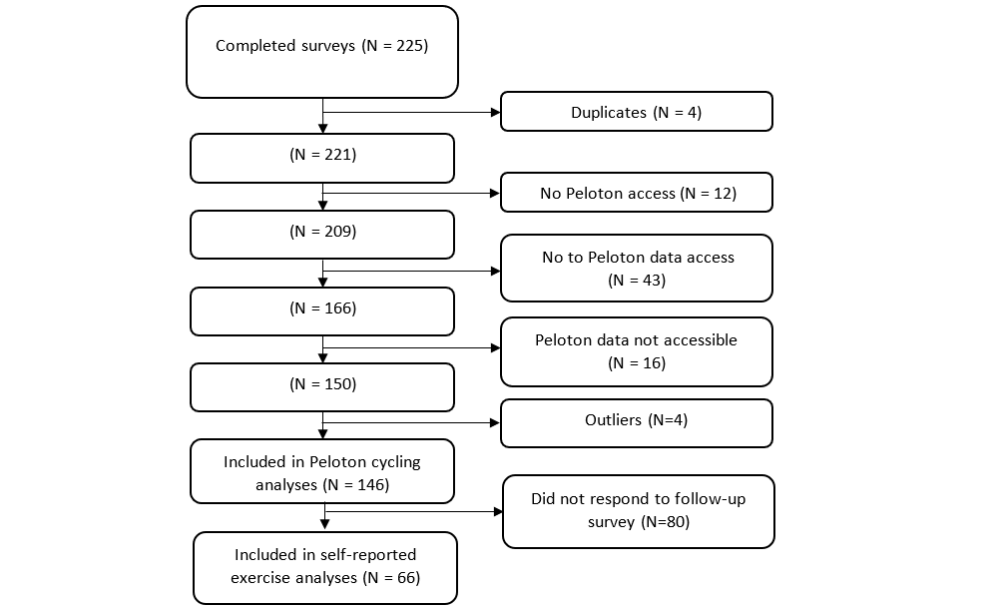
Exclusion criteria used to identify participants in the analyses.

Over the course of this 4-week period, 225 surveys were completed. Figure 1 illustrates the exclusion criteria that were used to identify the sample of 146 respondents included in this analysis using Peloton cycling data as the indicator of PA participation. Exclusion criteria were not having access to a Peloton Bike in their home, denying access to Peloton usage data, or not sharing their Peloton username. Four survey respondents were excluded from the analyses because they were outliers (ie, more than 3 SDs from the mean) on at least 2 key variables (eg, frequency and duration of Peloton cycling classes).

A follow-up survey request was sent via REDCap 12 weeks after each respondent completed the initial survey. The timing of the follow-up survey request was automated so that the request went out 12 weeks after the first survey was completed. To encourage responses, 2 additional reminder invitations were emailed at 3-day intervals after the first request. A total of 66 follow-up surveys were returned (response rate 45%).

Peloton usage data were accessed via the “follow” function of the Peloton app. After acquiring respondents’ permission, we used Peloton’s application programming interface (API) to access the exercise data of our survey participants. Using each participant’s Peloton username, we downloaded data from the riders’ first ever cycling class through 12 weeks after the initial survey was completed, which included the date and duration of each cycling class. For each participant, we defined 2 fixed dates for our analysis: the start date (starting date of using the Peloton Bike continuously) and the initial survey date. We defined the start date as the first date after which the user had at least 4 workouts within a 4-week period. Using this definition, we were able to adjust the start date to account for participants who may have set up a Peloton account for a single trial and then entered a period of no activity until they obtained their own Peloton Bike. We divided the participants’ activities into pre- and postsurvey activities in reference to the date on which the participant completed the initial REDCap survey.

### Measures

#### Demographics

For purposes of sample characterization, respondents were asked to self-report age, gender, race or ethnicity, the highest level of education completed, and whether they were a California resident.

#### Stress

##### Perceived Stress Scale

General perceived stress was assessed on both the initial and the follow-up surveys using the 10-item version of the Perceived Stress Scale (PSS) developed and validated by Cohen et al [30]. A review of studies validating this scale reported a Cronbach α greater than .70 in 11 out of 11 studies and a test-retest correlation of greater than .70 in 4 out of 4 studies [31]. Items were scored on a 5-point scale of *never* to *very often* with reference to the past month. The scale showed strong internal reliability in the present sample (Cronbach α=.86).

##### COVID-19 Stressors and Associated Perceived Stress

A modified version of a COVID-19 stress scale developed by Park et al [32] was used in the initial survey to gauge the level of impact that the COVID-19 epidemic had wrought on the study participants in the past month. Items on the Park et al [32] scale were constructed on the basis of prior work related to severe acute respiratory syndrome–associated coronavirus [33]. In this study, 8 “yes” or “no” items (see Table 1) asked whether the respondent had experienced a particular COVID-19–related situation in the past month. For each of the 8 COVID-19 stressors endorsed by a respondent, the level of associated perceived stress was rated by the respondent on a 5-point scale of *not at all stressful* to *extremely stressful*. A total perceived COVID-19 stress score was obtained by computing the mean of all the COVID-19 perceived stress scores. This scale has not been previously validated. In this study, the correlation between total perceived COVID-19 stress and general perceived stress (assessed using the PSS) was significant (*r*=0.46; *P*<.001).

**Table 1:** Participants’ self-reported COVID-19 stressors and associated perceived stress^a^ (N=146).

COVID-19 stressor items	Participants who answered yes, n (%)	Stressfulness rating, mean (SD)
Risk of becoming infected with COVID-19	83 (57)	3.18 (1.10)
Risk of loved ones becoming infected with COVID-19	102 (70)	3.44 (1.03)
Read or heard others talk about the severity and contagiousness of COVID-19	143 (98)	3.17 (1.09)
Uncertainty about how long quarantine or social distancing requirements will last	113 (77)	3.25 (1.07)
Changes to daily personal care routines (eg, cooking, cleaning, exercise or relaxation, and hobbies)	56 (38)	2.46 (1.14)
Changes to daily work routines (eg, unable to earn money or attend full- or part-time work schedule)	25 (17)	3.88 (1.05)
Cancellation of planned or scheduled celebrations, entertainment, vacations, or trips (eg, graduations, birthdays, and concerts)	99 (68)	2.90 (1.27)
Inability to travel (eg, cancelation of vacations and weekend trips)	97 (66)	2.77 (1.09)

^a^Responses were *yes* or *no* to the following prompt: “During the COVID-19 pandemic, many people have experienced unique sources of stress. Please indicate which of the following you have experienced in the past month. Stressfulness was rated on a 5-point scale (not at all to extremely).”

#### Exercise Behavior

##### Self-reported Exercise

On the REDCap survey, participants were asked to report their participation in exercise for 3 life stages: teenage, twenties, and now. Using the Godin Leisure-time Exercise Questionnaire (GLTEQ) [34] format, participants were asked to report how many times per week they engaged in (strenuous or moderate) exercise for more than 15 minutes in a typical week. These frequencies were then summed to yield the number of times per week that the participant engaged in moderate to vigorous exercise for at least 15 minutes. Self-reported exercise in a typical week (now) was also assessed on the follow-up survey 12 weeks after the initial survey. The GLTEQ has been validated against measured cardiorespiratory fitness [35] and as a means of distinguishing active from inactive adults [35].

##### Peloton Usage Data

Data retrieved from the Peloton API were used to estimate exercise behavior in the 4-week period prior to the initial survey and for the 12 weeks following the initial survey. For the 4-week period prior to the survey completion, a mean number of cycling days per week was computed as an indicator of frequency of exercise. Similarly, for the 12 weeks following survey completion, frequency was expressed as mean number of cycling days per week. The duration of cycling per week (both pre- and postsurvey) was calculated as the mean number of minutes per week.

### Statistical Analyses

Hierarchical regression equations were used to test the association between perceived stress and exercise. Parallel analyses were conducted first using estimates of exercise participation (frequency and duration) derived from the Peloton data on cycling during the 12 weeks after the survey (frequency expressed as mean times per week and duration expressed as mean minutes per week, respectively) and second using the self-reported data on moderate to vigorous exercise reported on the 12-week follow-up survey. Step 1 of each regression analysis controlled for age and gender. Step 2 controlled for exercise behavior at the time of the first survey (either Peloton cycling data for the 4 weeks prior to the survey or self-reported moderate to vigorous exercise obtained on the initial survey). Perceived stress (assessed using the PSS) was entered on step 3 of each analysis.

### Ethical Considerations

This study was determined to be exempt from human subject research ethics review. Study participants provided affirmative consent for the research even though there was a waiver of consent owing to data deidentification, public availability of the Peloton data, and usage of standard survey procedures. No compensation was provided to the study participants.

## Results

### Study Participants

Table 2 provides descriptive statistics for the 146 survey respondents included in the Peloton cycling analysis. Overall, the sample was majority female, highly educated, in good to excellent health, and about 70% non-Hispanic White. The majority of respondents (78%) were California residents. The age range was 22 to ≥70 years, with more than half of the respondents reporting an age between 30 and 49 years. Based on their self-reports (Table 3), participants represented an active sample, with the frequency of self-reported moderate to vigorous exercise being highest “now” (over 8 times per week) as compared to either frequency during teenage years (paired *t*_116_=4.23; *P*<.001) or that during their twenties (paired *t*_113_=5.04; *P*<.001). Exercise behavior was not significantly different between participants in their teens and those in their twenties (*P*=.43). A comparison of respondent characteristics between the full survey sample of 225 respondents and the subsample included in the Peloton cycling analysis (N=146) revealed no significant differences in demographics, stress levels, or self-reported exercise participation. A comparison of the study participants who completed the 12-week follow-up survey with those who did not revealed that the mean education level was higher among those who completed the second survey (t_144_=–2.63; *P*=.009). Among participants who did not complete the follow-up survey, 42% of them reported having a graduate degree, whereas among those who did complete the follow-up survey, 66% of them had a graduate degree. No other differences were found among the 146 participants in the Peloton data analysis and the 66 participants who completed the follow-up survey.

**Table 2:** Participant characteristics (N=146).

Characteristics		Participants, n (%)
**Gender**		
	Male	18 (12)
	Female	128 (88)
**Ethnicity**		
	Non-Hispanic White	99 (68)
	Hispanic	16 (11)
	Asian	14 (10)
	Black	16 (11)
	Decline to State	1 (1)
**Age (years)**		
	22-29	9 (6)
	30-39	44 (30)
	40-49	55 (38)
	50-59	28 (19)
	60-69	9 (6)
	≥70	1 (1)
**Education**		
	Some College	20 (14)
	College graduate	43 (30)
	Some Graduate	5 (3)
	Graduate Degree	78 (53)
**Health**		
	Fair	5 (3)
	Good	43 (30)
	Very Good	55 (38)
	Excellent	38 (26)
	Decline to State	5 (3)

**Table 3:** Participants’ self-reported exercise frequency (N=146).

Age group	Self-reported exercise frequency (times per week), mean (SD)		
	Moderate	Strenuous	Total
Teenage	3.31 (2.27)	3.45 (2.42)	6.95 (3.97)
Twenties	3.25 (1.24)	3.24 (2.32)	6.37 (3.67
Now	3.63 (2.29)	4.80 (1.73)	8.34 (3.06)

### Self-reported Stress

Responses to the items assessing COVID-19 stressors indicated that these were very prevalent (Table 1), with the most common stressors being hearing others talk about the risks of COVID-19 and uncertainty about how long quarantine or social distancing requirements might last.

Responses to the Cohen PSS indicated that the mean PSS score among participants (mean 25.27, SD 6.06) was on the high side of the “moderate” range, typically given as 14-26. Stress levels across the 2 surveys separated across 12 weeks were highly correlated (*r*=0.67; *P*<.001), suggesting that stress levels were fairly stable over time.

### Peloton Usage

Data from the Peloton API revealed that at the time of the initial survey, 25% of participants had been using the Peloton Bike for fewer than 4 months, and 25% of them had been using the Peloton Bike for more than 20 months (Table 4). The mean frequency of use during the 4 weeks preceding the survey was high, with 60% of the participants recording at least 1 cycling class on at least 5 days per week. The minimum duration of cycling during the 4 weeks preceding the survey was 20 minutes per week, and based on Peloton usage, 50% of study participants met or exceeded US recommended guidelines [36] for PA of 150 minutes per week of moderate to vigorous PA.

**Figure 2:**
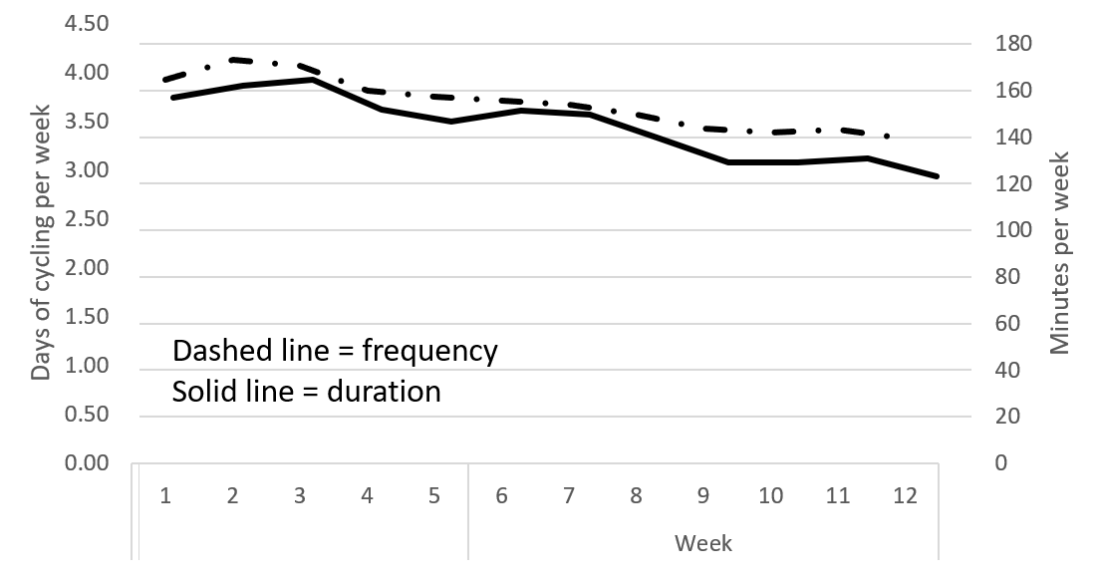
Frequency and duration of cycling decreased over the 12 weeks after the survey (N=146).

Figure 2 illustrates the pattern of Peloton cycling across the 12 weeks following the initial survey. Both frequency (days per week) and duration (minutes per week) decreased over time (*P*<.001 for both). In the week immediately following the survey, study participants completed a mean of 3.92 (SD 2.04) days of cycling and engaged in a mean of 156.89 (SD 108.77) minutes of cycling. During the last week of the 12-week period, participants completed a mean of 3.32 (SD 2.20) days of cycling and engaged in a mean of 122.98 (SD 97.67) minutes of cycling. During the final week of the 12-week monitoring period, 37% of participants met or exceeded 150 weekly minutes of cycling.

**Table 4:** Peloton application programming interface data showing that participants were overall habitually active (N=146).

Characteristic		Value
**Length of Peloton usage (weeks), n (%)**		
	<20	37 (25)
	21-38	35 (24)
	39-80	37 (25)
	81-288	37 (25)
**Peloton cycling during the 4-week presurvey, mean (SD)**		
	Frequency (times per week)	5.96 (3.13)
	Duration (minutes per week)	142.46 (73.23)

### Impact of Stress on Exercise Behavior

As gender was not significantly associated with Peloton cycling (frequency or duration) in any of the equations, gender was excluded, and the analyses were rerun with only age as a covariate in step 1. Age significantly predicted Peloton cycling duration (*R*^2^=0.05; *F*_1,144_=8.45; *P*=.004) and Peloton cycling frequency (*R*^2^=0.03; *F*_1,144_=4.49; *P*=.03). The model in step 2, in which Peloton usage during the 4 weeks prior to the survey was entered as a predictor variable, was a significant predictor of the frequency of postsurvey Peloton cycling (*R*^2^=0.57; *F*_2,143_=95.27; *P*<.001) and for predicting the duration of postsurvey Peloton cycling (*R*^2^=0.58; *F*_2,143_=102.58; *P*<.001). The change in *R*^2^ in step 2 suggested that presurvey exercise explained approximately 50% of the variance in postsurvey exercise. The regression models in step 3 of the 2 different equations examining the relationship of stress (assessed using the PSS) to postsurvey Peloton usage revealed that stress was not significantly associated with postsurvey cycling (change in all *R*^2^<0.003; *P* [duration of cycling]=.81; *P* [frequency of cycling]=.76).

In the regression analysis examining the impact of stress (assessed using the PSS) on overall self-reported exercise (assessed using the GLTEQ), neither age nor gender were associated with exercise at 12 weeks; hence, the analysis was performed without these 2 potential covariates. Overall moderate to vigorous exercise reported on the initial survey significantly predicted overall exercise reported on the 12-week follow-up survey (*R*^2^=0.31; *F*_1,64_=29.03; *P*<.001). There was no change in *R*^2^ values when stress was entered into the model, and stress was not significantly associated with overall exercise at 12 weeks (*P*=.28).

## Discussion

### Principal Findings

To the best of our knowledge, this is the first study analyzing data from a cohort of habitually active adults with access to internet-connected fitness equipment in their home, which demonstrated that exercise participation over a 12-week period was not predicted by reported stress levels. These data were collected during a lockdown period during the COVID-19 pandemic, and self-reports indicated that study participants were in fact experiencing a high prevalence of COVID-19–related stressors and a high level of perceived stress. Peloton cycling behavior over the 12 weeks following the initial self-report surveys was tracked through the Peloton cycling app and was hence not vulnerable to self-report bias. Although there was an overall trend for exercise participation to decrease over time in the study sample, stress was not significantly associated with the frequency or duration of postsurvey Peloton cycling. The absence of an impact of stress on exercise was confirmed in an analysis of the relationship of perceived stress with changes in self-reported overall moderate to vigorous exercise over the same 12-week period, as measured using the GLTEQ.

In contrast to the majority of studies investigating the impact of stress on PA or exercise, the usage of exercise participation data that were not obtained through self-report is a unique feature of this study. In their review of the topic, summarizing the findings of 168 studies, Stults and Sinha [16] identified only 2 studies that used objective PA assessments, and both of these studies were cross-sectional. The selection of research participants, all of whom had access to state-of-the-art home exercise equipment, is another defining feature of this study. The finding that among this self-selected sample of habitually active individuals, perceived stress did not negatively impact exercise participation is consistent with the findings of a few prior studies that relied on self-report to estimate exercise [23,24,37]. It is worth noting that all of these studies were conducted during the COVID-19 pandemic, reflecting the widespread concern that social distancing and stay-at-home orders would result in general declines in PA. Described by some as a “vicious circle” [38], in which social isolation begets increased stress, which leads to decreased PA and an associated decline in the ability to cope with stress, the type of chronic stress engendered by a global pandemic has the potential to have considerable cumulative and long-term health effects even among those who are not infected. Our study suggests that home-based fitness equipment may play a role in preventing declines in activity among those individuals who have already established an exercise habit. Future work should seek to disentangle the influence of the equipment access from the exercise habit.

Available data suggest that this sample of research participants was similar to the general population in terms of the amount of stress that they experienced during the COVID-19 pandemic. Overall, the prevalence of each of the COVID-19 stressors was comparable to that reported by Park et al [32] in a US sample assessed in April 2020, with the exception of changes to daily care personal routines and changes to work routines, both of which were far more prevalent in Park et al’s [32] study. This contrast may be a function of the timing of the data collection, as our study was conducted at least 8 months later into the pandemic than Park et al’s [32] study. Ratings of the perceived stressfulness associated with each COVID-19 stressor were comparable to the ratings reported by Park et al [32].

The fact that the individuals in this study were experiencing a relatively high level of stress overall is likewise supported by previously reported statistics. Cohen et al [39] reported on 3 large US surveys conducted in 1983, 2006, and 2009. In the study by Cohen et al [39], scores on the PSS were compared across demographic groups, with the highest mean score of 20.21 among unemployed men in 2006. More recently, a comparison of PSS scores across 1685 surveys deployed in 57 countries during the COVID-19 epidemic [40] yielded a global mean score of 19.08. With a mean PSS score of 25.27, the group of respondents in this study clearly perceived themselves as experiencing relatively high levels of stress.

During times of high stress, the health benefits of exercise may be even more salient than during times of low stress, so the identification of factors that support maintenance of exercise participation during times of stress has clear implications for public health. This study suggests that an established exercise habit may provide a buffer against the negative impact that stress typically has on exercise participation. The Peloton Bike is a stationary exercise bike that features a digital internet-connected monitor providing access to both live and on-demand cycling classes of varying duration and intensity. In addition to displaying continuous performance metrics, the monitor also shows a running list of other Peloton riders completing the same class and permits users to interact with other riders by sending out and receiving “high fives.” Since all of the study participants had access to this state-of-the-art fitness equipment, our results should not be generalized to persons who are engaging in other forms of exercise or those whose activity typically occurs outside the home. This type of fitness equipment, which combines accessibility with on-demand classes and connection to an internet-based community of exercisers, is relatively new and has not been extensively studied. There are many aspects of this technology that might encourage maintenance of an exercise program, including behavioral self-monitoring, competition, and social support for exercise.

In addition to the specific hypothesis tested in this analysis, our study demonstrates the considerable potential for internet-connected exercise equipment to be used to support both research and intervention. The ability to collect detailed data regarding exercise behavior enables personalized interventions, in which messaging could be delivered to individuals on the basis of their measured exercise participation. In terms of research, detailed information that can be obtained regarding the frequency and duration of exercise can facilitate many different avenues of research. Questions about the relationship of exercise participation to different health outcomes can be more rigorously explored using exercise data that are not biased by self-report, while questions about how best to support exercise adoption and maintenance can also be addressed using metrics of duration and frequency recorded by the exercise device. Although the focus on a single mode of activity (in this case, cycling) may limit the appeal to certain subgroups of individuals and may limit the generalizability of our research findings, as a model for conducting efficacy studies, internet-connected fitness equipment is an attractive option that merits further exploration.

### Limitations

Several aspects of this study may limit the extent to which the findings can be generalized outside of this particular sample. The participants in the study were overwhelmingly female and mostly non-Hispanic White, although approximately 30% of them reported affiliation with non-White ethnic or racial groups. Moreover, recruitment for the study was conducted through Peloton Facebook groups. Caution should be exercised, therefore, in generalizing our findings to men, non-White ethnic or racial groups, and persons who do not engage with social media. Another consideration is that the Peloton Bike only collects data when the Peloton Bike is being used. Therefore, there are other forms of activity (eg, walking, running, and swimming) that are not captured with this platform, which may have been differently affected by stress experienced during the COVID-19 lockdown. A possible explanation for the downward trend in Peloton Bike usage is that other forms of activity became available as COVID-19 lockdown restrictions were lifted. Nevertheless, even at the end of the 12-week monitoring period, the average duration of cycling was >2 hours per week, which is still sufficient to realize health benefits [41].

It should also be noted that the Peloton Bike functions as a subscription service; hence, in addition to the cost of the equipment, there is a monthly cost associated with accessing the live, on-demand classes. As such, it could be argued that our findings might not be generalizable to individuals who cannot afford this expense. Whether less affluent individuals with access to internet-connected home-based fitness equipment would exhibit a similar resilience to reductions in PA during times of elevated stress is an empirical question that could be explored in future research. There are already cases of health insurance companies providing access to the Peloton app free of charge to their members [42] and with the growing evidence that regular exercise lowers health care costs over the long term [13], it is reasonable to expect that home-based equipment may eventually be made available at low or no cost to some individuals. Moreover, it should be noted that data from the National Health Interview Survey [43] indicate that even among the more affluent subgroups, fewer than 50% of adults meet recommended guidelines for PA participation. Thus, even if the findings of this study were only generalizable to more affluent individuals, the potential impact in terms of numbers of people and likely health benefit is still quite large.

### Conclusions

To conclude, habitually active adults using internet-connected home fitness equipment maintained their exercise habits while experiencing high stress during the COVID-19 lockdown. This finding emerged using both the in-app cycling data from the Peloton Bike and self-reported moderate to vigorous leisure time activity. Given the strength of the evidence for the health benefits of exercise, identifying strategies for keeping individuals active during times of stress has tremendous public health implications. Future research should examine what role internet-connected home fitness equipment plays in supporting maintenance of exercise during periods of stress.

## Abbreviations

API: application programing interface
GLTEQ: Godin Leisure-time Exercise Questionnaire
PA: physical activity
PSS: perceived stress scale
REDCap: Research Electronic Data Capture

## Data Availability

The data sets generated or analyzed during this study are available from the corresponding author on reasonable request.
